# Human Papillomavirus (HPV)-Associated Primary Squamous Cell Carcinoma of the Rectosigmoid Colon: A Case Report and Literature Review of a Rare Malignancy

**DOI:** 10.7759/cureus.107650

**Published:** 2026-04-24

**Authors:** Mackenzie C Conner, Rutva Patel, Taija White, Youssef El Hamshary, Jordan Ditchek, David S Marshall, Jeffrey P Snow, Gary Schwartz

**Affiliations:** 1 Medicine, Nova Southeastern University Dr. Kiran C. Patel College of Allopathic Medicine, Davie, USA; 2 Radiology, Nova Southeastern University Dr. Kiran C. Patel College of Allopathic Medicine, Davie, USA; 3 Pathology, Memorial Healthcare, Hollywood, USA; 4 General Surgery, Memorial Healthcare System, Hollywood, USA; 5 Orthopedic Surgery, Nova Southeastern University Dr. Kiran C. Patel College of Allopathic Medicine, Davie, USA

**Keywords:** case report, chemoradiation, circulating tumor dna, complex surgical resection, hpv-related carcinoma, primary colonic squamous cell carcinoma, surgical resection

## Abstract

Primary colonic squamous cell carcinoma (SCC) represents a small minority of all colorectal cancer cases. Common clinical presentations include abdominal pain, weight loss, anorexia, dyschezia, and hematochezia. While adjuvant chemotherapy/radiation may be considered, treatment of colonic SCC is primarily surgical. We report a case of primary colonic SCC. A 66-year-old man with multiple comorbidities and a 42-pack-year smoking history presented with abdominal pain, constipation, and rectal bleeding. Colonoscopy findings included an anorectal ulcer and an ulcerated rectal mass, diagnosed as poorly differentiated, HPV-related SCC. Surgical treatment consisted of a low anterior resection with ileostomy and adjuvant chemoradiation, complicated by leukopenia/neutropenia. The ileostomy was reversed six months later with no gross malignancy. However, circulating tumor DNA (ctDNA) remained positive with a follow-up positron emission tomography scan and biopsy confirming recurrent SCC. The patient is currently receiving combination chemotherapy with carboplatin and paclitaxel, with considerations being made for cytoreductive surgery and hyperthermic intraperitoneal chemotherapy. This case demonstrates the role of ctDNA as an early marker of recurrence and an association with HPV infection. However, the risk factors for primary colonic SCC remain unclear, and adjuvant chemoradiation lacks consensus. Further evaluation of primary colonic SCC cases is necessary to determine standardized management guidelines.

## Introduction

Squamous cell carcinoma (SCC) classically arises in regions of the body that are lined with squamous epithelium. In the gastrointestinal (GI) tract, SCC arises more commonly in the anal canal and the esophagus [1,2]. In the colon, however, over 90% of malignancies are adenocarcinomas, which arise from glandular tissue [3]. Primary colonic SCC has been documented in the literature, although it is a rare cancer.

According to a 2022 case report on primary colonic SCC, this type of cancer represents less than 1% of all colorectal cancer (CRC) cases [4]. The first reported case of primary SCC of the colon was in 1919, but according to a recent case report on primary colonic SCC, approximately 150 cases have been reported in the literature since [5]. In addition to being a rare carcinoma, it is highly malignant and is characteristically poorly differentiated with high chances of metastasis.

There are various theories regarding the pathogenesis of SCC of the colon and rectum. One popular theory hypothesizes that injury to pluripotent stem cells in the colon may cause colonic SCC [4,6].

Common clinical presentations include abdominal pain, weight loss, loss of appetite, dyschezia, hematochezia, and changes in bowel habits [6]. Most literature reports that primary colonic SCC presents at a more advanced stage; according to one literature review, symptoms may also include anemia or a palpable mass [7]. Treatment options for colonic SCC focus on surgical resection. However, many studies have proposed the benefits of adjuvant chemotherapy and/or radiation therapy. In this report, we examined a case of primary SCC of the colon.

## Case presentation

**Figure 1:**
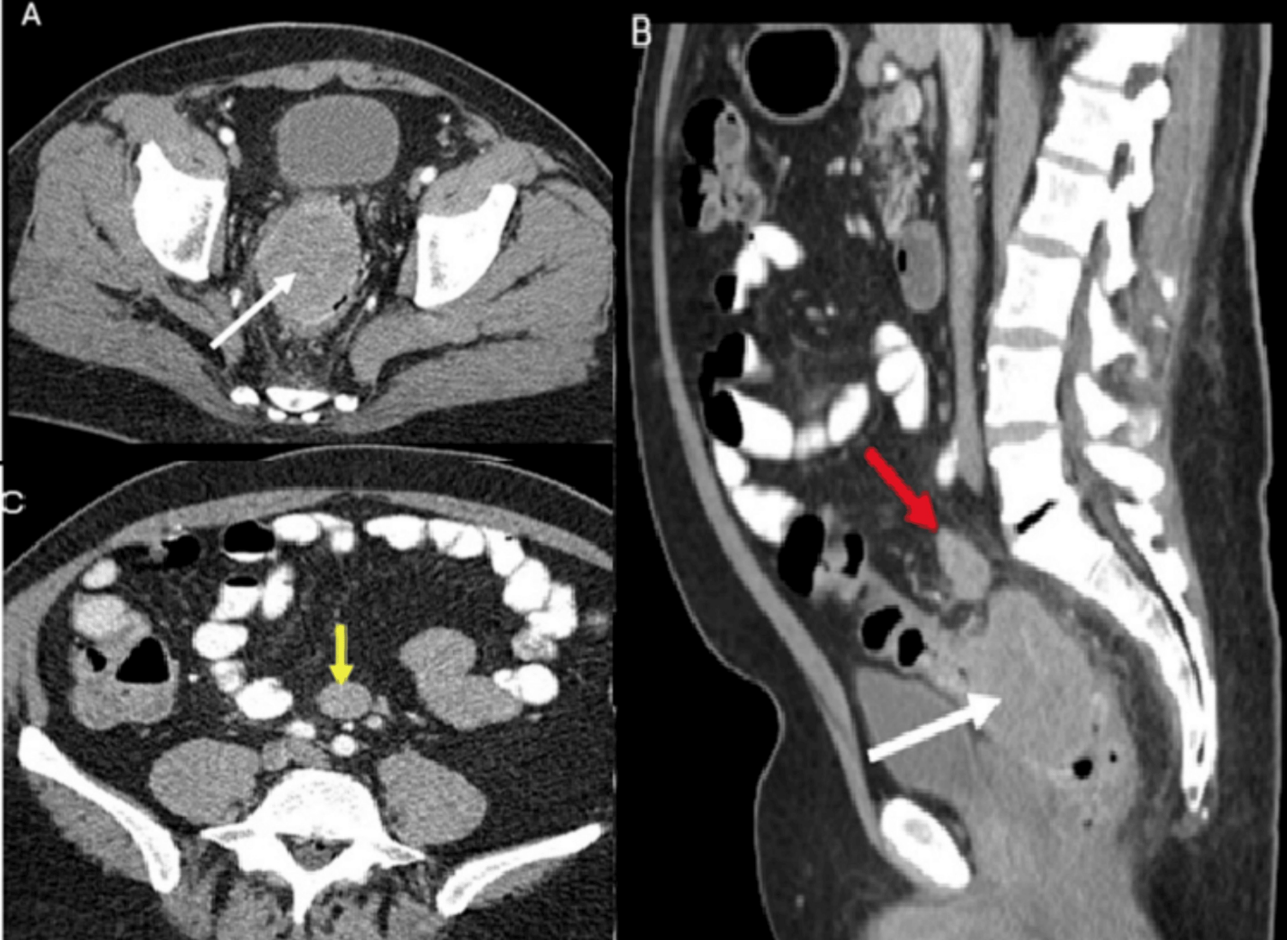
Preoperative CT scan of the abdomen and pelvis Axial (A) and sagittal (B) contrast-enhanced images of the abdomen and pelvis showing a large mass (7 x 5 cm; white arrows) centered in the right side of the proximal rectum. Note the nodal mass (red arrow) located just superior to the rectosigmoid mass. An axial image (C) obtained more superiorly shows an additional nodal mass (yellow arrow) in the lower retroperitoneum CT: computed tomography

A 66-year-old man with a 42-pack-year smoking history and a past medical history significant for hyperlipidemia, hypertension, type 2 diabetes, coronary artery disease, obstructive sleep apnea, and skin cancer was initially evaluated for several days of abdominal pain, constipation, and rectal bleeding. The patient has no personal or family history of colon cancer. The patient initially underwent abdominal/pelvic computed tomography (CT) scan to rule out the possibility of a small bowel obstruction. No obstruction or free air was seen, but a soft mass measuring 7.0 x 5.5 x 6.1 cm in the rectosigmoid junction with enlarged retroperitoneal lymph nodes was identified (Figure 1).

**Figure 2:**
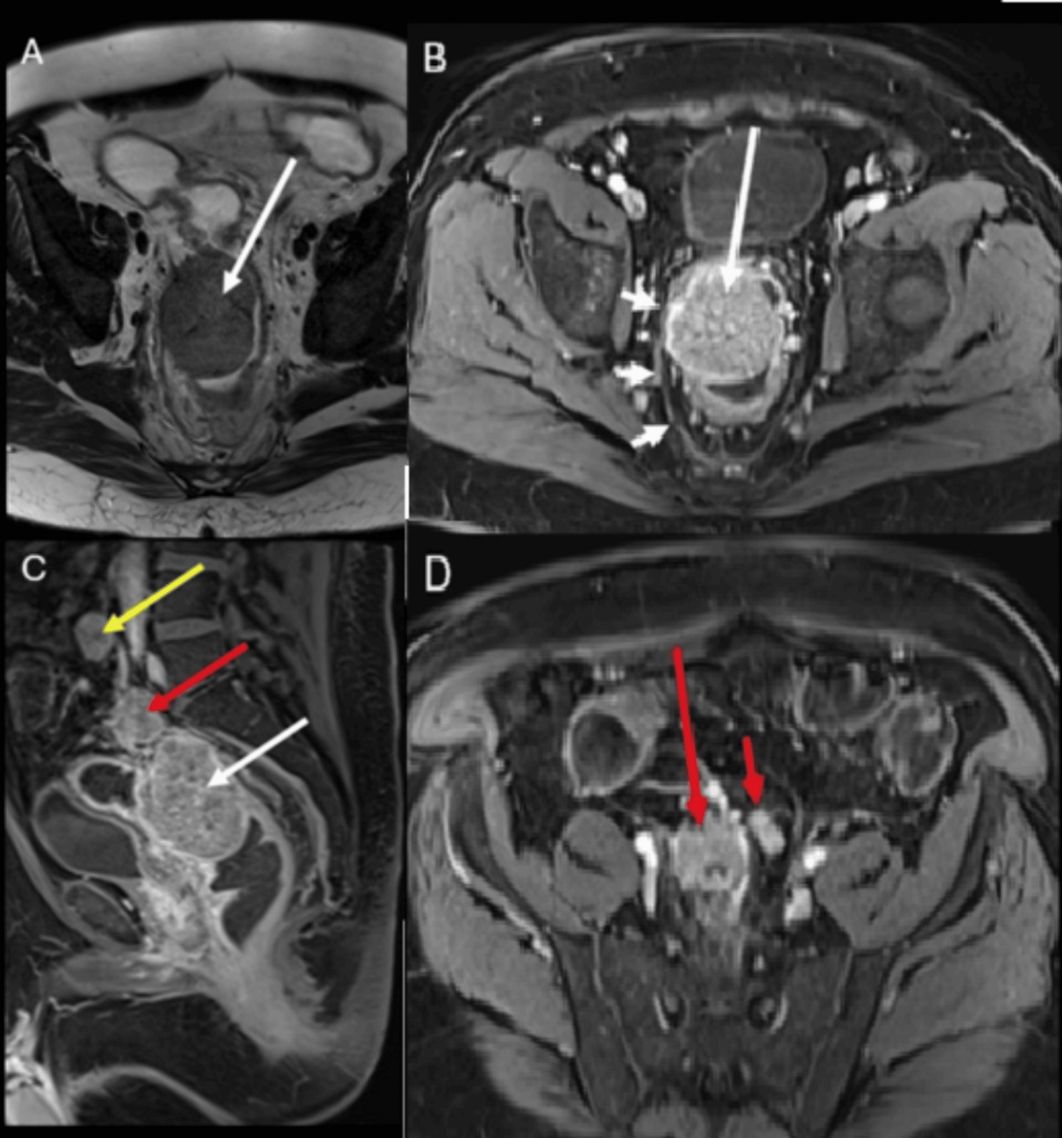
Preoperative MRI scan of the abdomen and pelvis Preoperative axial T2-weighted image (A) and axial contrast-enhanced T1-weighted image (B) of the rectum show the large mass (long white arrows) in the rectosigmoid colon. Note that the mass extends laterally on the right, where it is inseparable from the mesorectal fascia (short white arrows). A sagittal contrast-enhanced T1-weighted image (C) demonstrates the rectosigmoid mass (white arrow), a high mesorectal nodal mass (red arrow) just superior to it, and a low retroperitoneal (extramesorectal) nodal mass (yellow arrow) even more superior. An axial contrast-enhanced T1-weighted image obtained through the upper pelvis (D) shows the high mesorectal nodal mass (long red arrow) seen in figure (C) and a smaller enlarged lymph node (short red arrow) just to the left of it MRI: magnetic resonance imaging

A preoperative magnetic resonance imaging scan demonstrated a large mass in the rectosigmoid colon (Figure 2).

**Figure 3:**
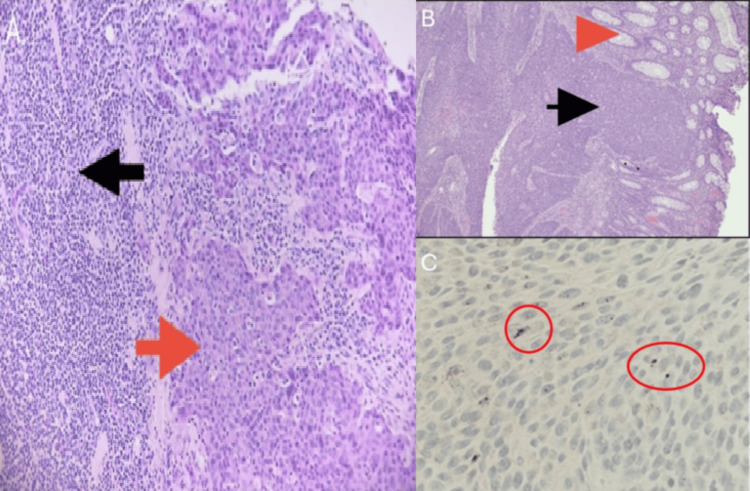
Histopathology of the rectosigmoidal mass Histopathology of the rectosigmoidal mass with H&E stain under high power (×20) (A) demonstrates benign glands/mucosa (red arrow) and tumor invading the perirectal soft tissue (black arrow). Under low power (×5) (B), it demonstrates squamous cell carcinoma (red arrow) invading the lymph node tissue (black arrow). Stained high-risk cocktail (×40) (C), which tests for 15+ types of HPV, including the most common tumor-associated subtypes: 16, 18, 26, 31, and 33. A specific subtype of HPV is not known in this case. The red circles point to small nuclear dots of signal, indicating HPV positivity H&E: hematoxylin and eosin; HPV: human papillomavirus

The patient followed up with a GI specialist and underwent a colonoscopy, which was changed to a sigmoidoscopy due to poor prep. During the procedure, the following were identified and biopsied for pathology: 1 cm superficial ulceration at the anorectal junction, a 5 mm polyp in the proximal rectum, and a large mass measuring 10 cm in length occupying the rectosigmoidal junction located 20 cm from the anal verge. The mass was malignant-appearing and friable, with superficial ulcerations and easy bleeding upon contact. Both samples were consistent with extensive involvement of human papillomavirus (HPV)-related invasive poorly differentiated SCC invading into the pericolorectal tissue. Sections revealed invasive SCC involving the upper rectum and distal sigmoid colon. The carcinoma involved the lamina propria and submucosa but did not appear to be arising from the luminal glandular epithelium. Immunohistochemical studies were performed for the rectosigmoidal mass and found that the tumor cells were strongly positive for keratin ⅚, p63, and p16 (Figure 3). The polyp in the proximal rectum was found to be a hyperplastic polyp with small, detached fragments of invasive poorly differentiated SCC.

Following diagnosis, the patient was admitted for surgical evaluation following two episodes of bright red blood per rectum with blood clots, associated mild dizziness with a white blood cell count of 14.5 x109 and hemoglobin of 12.3 g/dL. A colorectal surgeon was consulted and discussed the likelihood of needed adjuvant therapy following surgical resection. Surgery was scheduled to avoid further constipation issues due to tumor obstruction. A laparoscopic ileostomy was started but was converted to an open laparoscopic lower anterior resection and ileostomy with flexible sigmoidoscopy due to a narrow pelvic inlet and overall size and location of the tumor. This revealed a rectal mass attached to the right lateral sidewall, consistent with SCC HPV-associated perirectal tissue. The lower margin of the neoplasm and the anastomosis were located 8 cm from the anal verge. Positive radial margins were identified, and 2/23 lymph nodes were found to be pT3/ pN1b-positive. The composite stage was unknown. The patients' recovery was complicated by a paralytic ileus, which was treated by a nasogastric tube and maintaining NPO status. Further evaluation with a positron emission tomography (PET)/CT scan demonstrated increasing hypermetabolic retroperitoneal lymphadenopathy. A CT-guided biopsy was not feasible due to proximity to arterial structures. The patient was started on a combined treatment regimen of external beam radiation therapy using intensity-modulated radiation therapy targeting the pelvis/lower para-aortics and inguinal lymph nodes, and mitomycin C (5 mg/m^2^)/5-Fluorouracil chemotherapy for the treatment of the residual rectal SCC. The patient’s therapy was complicated by severe leukopenia and neutropenia. Further molecular testing demonstrated microsatellite-stable/mismatch repair-proficient status, high tumor mutational burden, and positivity for KRAS/NRAS/BRAF/WT/PD-L1. After six months of treatment, the patient returned to the surgical center, and an ileostomy reversal was performed; visual inspection of the abdomen appeared negative for malignancy. Signatera (Natera, Inc., Austin, TX) circulating tumor DNA (ctDNA) levels showed a downward trend over the following months but remained positive. At the beginning of treatment, the value was +184.8. Three months later, it decreased to +32.29, then to 26.26, and finally to +19.41 (normal values are undetectable). Five months after the ileostomy reversal, the patient returned for a follow-up PET/CT scan. This demonstrated a new area of proximal sigmoid colon hypermetabolic wall thickening, and a new hypermetabolic left lower quadrant 1 cm nodule. Radiological imaging suggested residual and/or recurrent disease, and the patient was scheduled for a colonoscopy. This revealed erythema, congestion, friability, and ulceration at the descending colon anastomosis. Biopsies taken during the procedure were consistent with SCC. A follow-up PET/CT scan demonstrated stable proximal sigmoid colon wall thickening and an adjacent hypermetabolic 1 cm left pericolonic gutter nodule. The patient was started on a combination of carboplatin/paclitaxel systemic chemotherapy for the recurrent rectal mass, with consideration of cytoreductive surgery ± hyperthermic intraperitoneal chemotherapy in the future based on the patient's response to therapy. The number of cycles and doses is unknown. Regarding the Eastern Cooperative Oncology Group Performance status, the patient was restricted in strenuous activity but was ambulatory, corresponding to a level 1 on the scale. A visual timeline of the patient’s symptoms, evaluation, surgery, and recovery is seen in Table 1.

**Table 1:** Timeline of the patient’s clinical course This timeline shows the sequence of events starting from the initial presentation until one year after surgery MMC: mitomycin C; 5FU: 5-fluorouracil

Day	Event
0	Patient presents with bright red blood per rectum
2	Patient admitted to hospital; general surgery consult; oncology consult
6	Surgery performed
69	Tumor board evaluation
87	Radiation oncology evaluation
102	Began chemotherapy: MMC/5FU/D29; began radiation therapy: 50.4 Gy, 28 fractions
165	Radiation was completed
251	Ileostomy was reversed
252	Colonoscopy for recurrent disease; tumor board evaluation; began chemotherapy (carboplatin/paclitaxel)
1 year	Continued follow-up

## Discussion

Primary SCC of the colon has also been associated with viral and parasitic infections. For example, there has been a reported case of primary colonic SCC in a patient with no personal or family history of colon cancer that was theoretically linked to a persistent *Entamoeba histolytica* infection that did not resolve with emetine hydrochloride treatment [8]. There have also been cases where a link between HPV and primary SCC of the colon has been reported. This theory of pathogenesis involves the classic E6/E7 oncogenes of HPV, which inhibit the tumor-suppressor genes p53 and Rb, respectively [9]. As a result, uncontrolled viral proliferation within the infected host leads to neoplasia of the colonic mucosa [10]. Another association reported in the literature is between idiopathic inflammatory bowel disease (IBD) and primary SCC of the colon and rectum [11]. Reported cases often present a patient with chronic IBD, for example, ulcerative colitis, that is complicated by SCC of the colon [11].

In this report, we examined a case of primary SCC of the colon. In this case, ctDNA was used as a marker of recurrence. Ct(DNA) is released into the bloodstream by tumor cells, which makes it a useful tool for determining factors such as tumor progression and prognosis of disease [12].

In addition to being a single-patient observation, this case report has several limitations that restrict the generalizability of its findings to guide standardized management for primary colonic SCC. One significant limitation is the difficulty in definitively classifying the tumor as a primary colonic vs. anal SCC. Given its location only 20 cm from the anal verge, there remains a possibility that the malignancy originated from the anal canal and extended proximally into the colon. Additionally, although HPV association, as seen in this case, is a well-established risk factor for anal SCC, its link to colonic SCC remains unclear.   Some of the initial symptoms can overlap with benign conditions, such as hemorrhoids, diverticulitis, and typical colorectal adenocarcinomas.

Another limitation was the inability to perform a CT-guided biopsy of retroperitoneal lymph nodes due to their proximity to arterial structures. The lack of histologic confirmation of suspected metastatic disease introduces uncertainty regarding staging accuracy, prognosis, and assessment of treatment response. Furthermore, the follow-up duration was relatively short, restricting evaluation of long-term outcomes such as disease-free survival, overall survival, and ctDNA trends over time. The lack of HPV genotyping to confirm subtype 2, as well as this study being a single-institution experience with no comparison group, is a limitation of this study.

On the premise of monitoring, this case demonstrates the role of ctDNA as an early marker of recurrence, a promising surveillance tool to supplement radiographic evidence. Research shows that ctDNA can predict recurrence of colorectal adenocarcinoma with a median interval of 8.7 months earlier than radiography [13]. As such, it is evident why ctDNA is currently used as a valuable tool for predicting tumor burden and treatment response for adenocarcinoma of the colon [13]. Further research is indicated to determine whether ctDNA may be similarly predictive of outcomes in SCC of the colon.

Current treatment of primary colonic SCC is based on treatment protocols for adenocarcinoma of the colon, as there are no established guidelines for SCC of the colon. Additionally, there is a lack of consensus regarding adjuvant chemotherapy. In this case, adjuvant chemotherapy with carboplatin and paclitaxel was administered. According to a 2024 systematic review on treatment modalities for SCC of the colon, the most common treatment was surgery alone (52.1%), while only 12.9% of cases received adjuvant chemotherapy [14]. According to the study, patients treated with surgery and adjuvant chemotherapy had a statistically significant improvement in overall survival [14]. As such, further evaluation of cases and potential clinical trials are recommended to establish a suitable standard of care for patients with primary colonic SCC.

The rarity of primary colonic SCC often leads to delayed recognition, misdiagnosis, and underestimation of the aggressiveness of the disease. It is crucial to keep SCC of the colon as a differential because it has a poor prognosis [15]. This may be associated with the fact that SCC of the colon is often diagnosed at a later stage [16]. Additionally, SCC of the colon often is associated with metastases and local lymph node involvement [16]. Comparing the overall survival of adenocarcinoma and the SCC of the colon, SCC had a statistically significant association with poorer survival [17]. More vigilant consideration of the possibility of primary colonic SCC may aid in earlier diagnosis and better prognosis.

## Conclusions

Primary colonic SCC is a rare malignancy with unclear pathogenesis. Studies have proposed HPV infection as a potential contributor. This case describes colonic HPV-associated SCC treated with surgical resection in conjunction with radiation and chemotherapy. The case highlights the aggressive nature of the condition, diagnostic challenges encountered, and therapeutic obstacles encountered due to a lack of treatment guidelines. Given the recurrence of the malignancy in this case, the effectiveness of adjuvant chemotherapy remains unclear. This highlights the need for standardized management guidelines. This case also demonstrated the role of ctDNA as an early marker for surveillance and detection of recurrence. However, further investigation is needed to evaluate risk factors, pathogenesis, prevalence, and diagnostic guidelines.
